# Towards rapid genotyping of resistant malaria parasites: could loop-mediated isothermal amplification be the solution?

**DOI:** 10.1186/1475-2875-13-237

**Published:** 2014-06-16

**Authors:** Rashad Abdul-Ghani

**Affiliations:** 1 Department of Parasitology, Faculty of Medicine and Health Sciences, Sana’a University, Sana’a, Yemen

**Keywords:** Loop-mediated isothermal amplification, *Plasmodium*, Drug resistance, Genotyping, Point-of-care testing

## Abstract

Loop-mediated isothermal amplification (LAMP) is an innovative molecular technique that has been validated for point-of-care testing to diagnose malaria. Molecular detection and tracking of anti-malarial drug resistance is mainly based on highly sophisticated, costly and time-consuming techniques. With the validation of resistance-associated gene mutations in malaria parasites, there is a need to develop rapid, easy-to-use molecular tests for anti-malarial drug resistance genotyping. LAMP could be further developed as a point-of-care test to rapidly detect anti-malarial drug resistance-associated molecular markers, thereby help detecting and monitoring drug resistance in surveillance studies.

## Background

Resistance to anti-malarial drugs is one of the main obstacles to the control and elimination of malaria, leading to the resurgence of malaria incidences and deaths [1,2]. Molecular biology advances have made it possible to rapidly amplify and detect nucleic acids. The validation of point mutations in certain parasite genes has contributed to the application of these markers as tools for the detection and surveillance of anti-malarial drug resistance [3]. The detection of these markers relies basically on polymerase chain reaction (PCR) followed by detection using complicated approaches, including sequencing and restriction fragment length polymorphism.

The development of loop-mediated isothermal amplification (LAMP) was a major contribution in the rapid, accurate and cost-effective DNA amplification and detection [4,5]. LAMP has several characteristics that make it suitable as a point-of-care test (POCT) for malaria diagnosis. These include high sensitivity and specificity in diagnosis of malaria parasites, isothermal amplification, visual detection of products, stability of its reagents against blood inhibitors, simplicity of its procedure and reasonable cost [6]. It is likely that POCTs for nucleic acid detection of pathogens will serve an important tool for global health [7]. Myers *et al.*[7] designed an inexpensive, handheld, battery-powered point-of-care system to enable pathogen genotyping in the developing world. This opens new horizons for involving this technology in devising new tools for rapid genotyping of pathogens, including *Plasmodium* species. Several LAMP-based assays have been tested and evaluated for the detection of malaria parasites, with high levels of sensitivity and specificity being reported [6]. LAMP combined with DNA filter paper (FTA card) and melting curve analysis has been evaluated as a novel molecular system for diagnosis of falciparum malaria [8]. In addition to its high sensitivity and specificity compared to microscopy, this system reduces the risk of contamination due to the elimination of the need for gel electrophoresis [8].

One of the major limitations of LAMP compared to PCR is the complexity of primer design. LAMP requires the design of a set of at least two primer pairs to identify six regions of the target gene to increase the efficiency of the reaction, with the possibility of using two additional loop primers to accelerate the reaction [4,9]. The use of such a complex set of primers leads to the yield of a considerably higher amount of DNA compared to that produced by PCR. Therefore, such a complex set of primers makes LAMP more sensitive in detecting the target gene and more efficient in its amplification compared to conventional PCR. Despite being complex, LAMP primer design has been made easier with the availability of specific primer designing tools and software, including PrimerExplorer (Eiken Chemical Co., Tokyo, Japan) and LAVA [10].

## LAMP-based POCTs for malaria diagnosis

Abdul-Ghani *et al. *[6] have recently suggested the possibility to introduce POCTs for the diagnosis of malaria parasites in clinical and epidemiological settings based on LAMP principle. Following the suggestion of Abdul-Ghani *et al. *[6], several studies evaluated simplified LAMP-based kits for the diagnosis of malaria parasites [11-14]. Successful application of LAMP kits, consisting of plastic reaction tubes containing thermostable reagents, specific for *Plasmodium falciparum* DNA amplification has been reported with high sensitivity in a remote clinic in Uganda [11]. LAMP-Tube scanner is a rapid, inexpensive LAMP-based method developed by Surabattula *et al. *[12] as a portable device to diagnose *P. falciparum* and *P. vivax* with high sensitivity and specificity. Polley *et al.*[13] evaluated a LAMP malaria test kit for the diagnosis of imported falciparum malaria. The authors reported that this new kit was as accurate as nested PCR and was superior to expert microscopy, with a great reduction in performance time [13]. In addition, real-time LAMP (RealAmp) has been recently developed for rapid amplification and detection of *P. vivax *[14].

## Could LAMP-based POCTs be developed for anti-malarial drug resistance genotyping?

Recent reports showed the possibility to develop LAMP-based assays to detect resistance genes in bacteria [15-17]. This opens horizons for the application of this technique in detecting molecular markers of resistance in other pathogens. With the successful innovation of rapid, molecular LAMP-based kits for the diagnosis of malaria parasites, is the way now paved towards the rapid molecular detection of resistant *Plasmodium* genotypes? Being successfully deployed as POCT devices to detect and differentiate *Plasmodium* species, development and evaluation of LAMP-based POCTs to detect the molecular markers of anti-malarial resistance could be possible in the near future. This could be prompted for those validated molecular markers of resistance and would encourage the detection and/or validation of other markers, broadening the range of resistance monitoring. The low cost, turnaround time and technicality required for other molecular techniques make LAMP promising to develop rapid resistance genotyping kits that could be used for surveillance studies in resource-limited, malaria-endemic countries.

Innovation of LAMP-based POCTs for detecting resistant genotypes of malaria parasites could be an effective approach to facilitate anti-malarial resistance detection and tracking in resource-limited countries afflicted with the disease. Forwarding research towards the development of such POCTs should be prioritized, particularly with the widespread resistance to anti-malarial drugs and combinations.

## Competing interests

The author declares that he has no competing interests.
